# What Are the Health Benefits of Active Travel? A Systematic Review of Trials and Cohort Studies

**DOI:** 10.1371/journal.pone.0069912

**Published:** 2013-08-15

**Authors:** Lucinda E. Saunders, Judith M. Green, Mark P. Petticrew, Rebecca Steinbach, Helen Roberts

**Affiliations:** 1 Faculty of Public Health and Policy, London School of Hygiene and Tropical Medicine, London, United Kingdom; 2 General and Adolescent Paediatrics Unit, UCL Institute of Child Health, London, United Kingdom; University of Granada, Spain

## Abstract

**Background:**

Increasing active travel (primarily walking and cycling) has been widely advocated for reducing obesity levels and achieving other population health benefits. However, the strength of evidence underpinning this strategy is unclear. This study aimed to assess the evidence that active travel has significant health benefits.

**Methods:**

The study design was a systematic review of (i) non-randomised and randomised controlled trials, and (ii) prospective observational studies examining either (a) the effects of interventions to promote active travel or (b) the association between active travel and health outcomes. Reports of studies were identified by searching 11 electronic databases, websites, reference lists and papers identified by experts in the field. Prospective observational and intervention studies measuring any health outcome of active travel in the general population were included. Studies of patient groups were excluded.

**Results:**

Twenty-four studies from 12 countries were included, of which six were studies conducted with children. Five studies evaluated active travel interventions. Nineteen were prospective cohort studies which did not evaluate the impact of a specific intervention. No studies were identified with obesity as an outcome in adults; one of five prospective cohort studies in children found an association between obesity and active travel. Small positive effects on other health outcomes were found in five intervention studies, but these were all at risk of selection bias. Modest benefits for other health outcomes were identified in five prospective studies. There is suggestive evidence that active travel may have a positive effect on diabetes prevention, which may be an important area for future research.

**Conclusions:**

Active travel may have positive effects on health outcomes, but there is little robust evidence to date of the effectiveness of active transport interventions for reducing obesity. Future evaluations of such interventions should include an assessment of their impacts on obesity and other health outcomes.

## Background

The link between physical activity and health has long been known, with the scientific link established in Jerry Morris' seminal study of London bus drivers in the 1950s [1]. There is also good ecological evidence that obesity rates are increasing in countries and settings in which ‘active travel’ (primarily walking and cycling for the purpose of functional rather than leisure travel) is declining [2], [3]. Given that transport is normally a necessity of everyday life, whereas leisure exercise such as going to a gym may be an additional burden, and is difficult to sustain long term, [4], [5] encouraging ‘active travel’ may be a feasible approach to increasing levels of physical activity [6]. It is therefore plausible to assume that interventions aimed at increasing the amount of active travel within a population may have a positive impact on health. This has been the underlying rationale for recent public health interest in transport interventions aiming to address the obesity epidemic and a range of other health and social problems [7]; for example, *“For most people, the easiest and most acceptable forms of physical activity are those that can be incorporated into everyday life. Examples include walking or cycling instead of travelling by car, bus or train”* [8]. Active travel is seen by policy makers and practitioners as not only an important part of the solution to obesity, but also for achieving a range of other health and social goals, including reducing traffic congestion and carbon emissions [9].

It has been recommended that the public health community should advocate effective policies that reduce car use and increase active travel[10]. One recent overview concluded that active travel policies have the potential to generate large population health benefits through increasing population physical activity levels, and smaller health benefits through reductions in exposures to air pollution in the general population [6]. However, while a systematic review [11] has found that non-vigorous physical activity reduces all-cause mortality, the two studies which looked at active commuting alone [12], [13] found no evidence of a positive effect. There are a number of reasons why active travel may not contribute to overall physical activity levels. Some studies of young children have found no differences in overall physical activity levels for active and non-active commuters [14], [15], [16], perhaps because the distance walked to school may simply be too short to make a significant contribution. For both children and adults, it is unclear how far individuals may offset the extra effort of cycling or walking with additional food intake, or by reducing physical activity in other areas of everyday life. Additionally, there is evidence that the health benefits of exercise are not shared equally across populations, with the cultural and psychological meanings of activities such as walking or cycling potentially influencing their physiological effects [17], [18].

A reliable overview of the strength of the scientific evidence is therefore needed because the causal pathways between active travel and health outcomes such as obesity are likely to be complex, and promoting active travel may have unintended adverse consequences [19], for example by reducing leisure activity.

Existing studies show a mixed picture on the relationship between active travel and health outcomes including obesity [20]. Recent systematic reviews have focussed almost exclusively on cross-sectional studies [20], [22], [23], or one narrow health outcome [24] or on combined leisure and transport activity [25]. Obesity is a particular focus because the rise in the prevalence of obesity over the past 30–40 years has occurred in tandem with the decline of active travel, and overweight and obesity are now the fifth leading risk for death globally as well as being responsible for significant proportions of the disease burden of diabetes (44%), ischaemic heart disease (23%) and some cancers (7–41%) [21].

Given the widespread promotion of active travel for reducing obesity in particular, and improving the public health in general, it is perhaps surprising that is, to date, no clear evidence on its effectiveness. To address this gap, a systematic review of evidence from empirical studies was carried out with the objective of assessing the health effects of active travel specifically (rather than of physical activity in general, where the evidence is already well-established). This review was undertaken to identify and synthesise the relevant empirical evidence from intervention studies and cohort studies in which health outcomes of active travel have been purposively or opportunistically measured to assess the impact of active travel on obesity and other health outcomes.

## Methods

Eleven databases were searched for prospective and intervention studies of any design (Cochrane Library, CINAHL Plus, Embase, Global Health, Google Scholar, IBSS, Medline, PsychInfo, Social Policy and Practice, TRIS/TRID, Web of science – full details in Table 1). The review protocol is available on request from the authors. The search strategy adapted the search terms developed by Hoskings et al. [26] (2010 Cochrane Review) and Bunn et al. [27] (2003) to create a master search strategy for Medline (see Appendix S3) which was then adapted as needed to fit each database (The exact search strategy used in each database is available from the corresponding author). No time, topic or language exclusions or limits were applied. Hand-searching of relevant studies was also conducted, and bibliographies of identified papers were checked along with those of papers already known to the researchers. The PRISMA flow chart, PRISMA checklist and search strategy are included in Appendices S1, S2, and S3 respectively.

**Table 1:** The search strategy was conducted on the following databases.

Database	Total number of search results extracted	Date search results were extracted
Embase	6,497	13/08/10 & 09/11/12
Global Health	1,372	12/08/10 & 09/11/12
Medline	5,005	12/08/10 & 09/11/12
PsychInfo	718	12/08/10 & 09/11/12
Social Policy and Practice	38	12/08/10 & 09/11/12
IBSS	74	13/08/10 & 09/11/12
Web of Science	5,141	12/08/10 & 09/11/12
Cochrane Library	113	16/08/10 & 09/11/12
TRIS	162	13/08/10
TRID	301	09/11/12
CINAHL*	1960	18/08/10 & 09/11/12
Google Scholar*	848	03/09/10

* Results were checked by 1 reviewer and no new papers that had not previously been identified through handsearching and database searches were identified.

Two reviewers independently identified potentially relevant prospective studies. If it was not clear from the title and abstract whether the article was relevant to active travel, then the paper was reviewed in detail. Non-English language studies were eligible for inclusion, though no relevant studies were identified. One reviewer then screened the articles using the following inclusion criteria:

1. Prospective study examining relationship between active travel and health outcomes; or study evaluating the effect of an active travel intervention; and
2. Active travel (walking or cycling for transport rather than work or leisure) measured in a healthy population (e.g. using self report measures, or use of pedometers); and
3. Health outcome included.

Retrospective and single cross-sectional studies (e.g. one-off surveys) were excluded.

One reviewer extracted data including information on methods, outcomes (as adjusted relative risks, or hazard ratios; if these were not available or calculable, other effect measures were extracted – e.g. mean changes), populations and setting for each study. The quality assessment was conducted using a standardized evaluation framework, the ‘Evaluation of Public Health Practice Projects Quality Assessment Tool’ (EPHPP) al. [28] [29]. Two reviewers independently reviewed each study and discussed any differences to produce consensus scores for each study against each quality criterion (see Table 4).

## Results

Twenty-four studies reported in thirty-one papers were included (see Tables 2 and 3). Five were prospective cohort studies with obesity-related outcomes, all in children; fifteen were prospective cohort studies with other health outcomes; and five were intervention studies with other health outcomes (details of excluded studies available on request from the authors). For the prospective cohort studies the results are presented adjusted for covariates. There was variation in what adjustments were made by different studies but the adjustments did not have large impacts on effect size. Details of the methodological assessment of each paper are included in Table 4.

**Table 2:** Experimental and observational studies of active travel and health outcomes.

Author (Year) and Setting	Methods	Population	Results
**Intervention Studies**			
**De Geus et al. (2007)**Oost-Vlaanderen, Belgium	Trial to assess effects of active travel on fitness; 10 men and 8 women passive travellers selected and matched for sex and age; asked to cycle minimum of 2 km each way 3 days a week.Measurements at baseline, 12 and 24 weeks: Fitness test – measured maximal heart rate and oxygen consumption.	Aged 33–5444% women	Cycle commuting showed significant improvements in fitness after 12 weeks as measured by absolute and relative maximal power and maximal exhaustion.
**De Geus et al. (2008, 2009)**Oost-Vlaanderen, Belgium	Controlled trial to assess effects of active travel on fitness and cardiovascular health; 92 participants; 74 passive commuters (men and women) asked to cycle to work at least 3 times a week. 18 controls commuted as usual. 87% completion rate. Compliance of 38% in first 6 months and 34% in the second 6 months. Travel diary and distance recorder on bicycles measured activity. Measurements at baseline, 6 and 12 months: BMI; Fitness test – maximal external power and peak oxygen uptake; Overall activity levels; Blood pressure; Cholesterol; QOL; Leisure-time physical activity	Intervention GroupMean age 43 (+/−5 SD)BMI 26 (+/−3.8 SD)Control GroupMean age 49 (+/−7 SD)BMI 24.9 (+/−2.9 SD)	Minutes and calories burned per week through all physical activity were higher in the intervention group than the control group (but not statistically significant for minutes in the second 6-month period).
**Hendriksen et al. (2000)**Amsterdam, Netherlands	RCT to assess effects of active travel on fitness and BMI; 122 participants randomised and stratified for age and sex.Minimum intervention group participation was 3 km each way three times a week for 6 months. After 6 months the control group could commence cycle commuting at any frequency or distance they chose. 94% completion; after 1 year 13 had dropped out (11%).	Sedentary workers of 2 companies. Aged 25–56.29% womenIntervention Group: Mean age:Male 38.1 (+/−6.3 SD) range 26–56; Female 37.1 (+/−6.3) range 27–48; BMI: Male 25 (+/−2.3 SD) range 20–31; Female 26 (+/−4.6 SD) range 20–37Control Group: Mean age:Male 38.6 (+/−6.4 SD) (25–54); Female 36.3 (+/−6.9 SD)(29–49); BMI: Male 24 (+/−3.1 SD)(20–35); Female 25 (+/−4.7 SD) (18–36)	No significant weight change in control or intervention group after 1 year.Maximal external power increased in the intervention group 13% in the first 6 months while it stayed the same in the control group.Maximal oxygen uptake – significant change in men only in intervention group in first 6 months.
**Mutrie et al. (2002)**Glasgow, UK	RCT to assess effect of promotional pack on active travel. 295 participants; 89% participation; Participants not blind; 66% response rate at 6 months; Control group given intervention to encourage active travel after 6 months.	Employees at 3 public sector workplaces64% women;Mean age 38 (range 19–69);76% in social classes 1 & 2	3 of 8 SF36 subscales significantly improved in the mean intervention group score compared with the control group: Mental Health (72 to 76 vs. 73 to 71); Vitality (57–64 compared with 61); General Health (71 to 76 vs 75 to 73)
**Oja et al. (1991, 1998)**Finland	RCT to assess effects of active travel on various health outcomes;160 eligible volunteers selected from 860 participants in a postal survey71 passive commuter participants;96% participation; 10 weeks intervention group active commuting (mean 2.4 km walk or 9.7 km cycle), control group passively travelling. Followed by 10 weeks both groups actively travelling; Intervention compliance - 78% of workdays; Control compliance - 92% of workdays.	44% women.Intervention Group: Mean age: Male 41.7 (+/−7.2 SD)Female 38.4 (+/−8.2 SD)BMI: Male 25.1 (+/−2.7 SD); Female 24.4 (+/−3.5 SD); Control Group: Mean age: Male 40.5 (+/−7.6 SD)Female 38.4 (+/−8.4 SD)BMI: Male 25.7 (+/−2.4 SD); Female 24 (+/−3.9 SD)	4.5% (p = 0.02) net increase in maximal oxygen uptake in intervention vs control group and 10.3% net increase in maximum treadmill time (p = <0.001) and 5% (p = 0.06) increase in HDL cholesterol.No significant changes in serum total cholesterol or triglyceride concentrations.No changes in bodyweight or leisure-time physical activity in either group.
**Prospective Cohort Studies**			
**Andersen et al. (2000)**Copenhagen County, Denmark	Prospective Cohort Study to assess association between active travel & all-cause mortality. 13375 women, 17265 men randomly selected, followed prospectively for average 14.5 years. Uses pooled data from 3 population surveys conducted in 1964, 1970 & 1971, 1976 & 1978; registered deaths to 1994. Bicycling to work reported by 783 women, 6171 men (average 3 hours/wk).	Aged 20–9344% women	Relative risk of all-cause mortality of 0.72 (95% CI 0.57–0.91) in cycle-commuters compared to non-cyclists.Adjusted for age, sex, education, leisure time physical activity, BMI, blood lipid levels, smoking and blood pressure.
**Barengo et al. (2004)**Eastern & South-west Finland	Prospective Cohort Study; 16,824 women and 15,853 men drawn from independent random sample of national population register. Participation rate: Men 71–94% Women 78–95%6 cross-sectional surveys in 1972,1977, 1982, 1987, 1992, 1997 included: Self-administered questionnaire on physical activity behaviour in a typical week and assessing risk factors for CVD; Height, weight and blood pressure measured by a nurse and blood sample taken for serum cholesterol. Median follow-up 20 years.(13–25 years inter-quartile range)	Aged 30–5951% womenMean age: Men 43.4 (SD 8.4); Women 43.8 (SD 8.5);BMI 25–29.9:Men 47.2%; Women 34.9%Active travel to and from work:Men Women<15 mins 64.3 54.315–29 17 20.130+ 18.7 25.6	Adjusted hazard ratio (95% CI)All cause mortality:Men:<15 min 1.0015–29 min 1.01 (0.92–1.11)30+ min 1.07 (0.98–1.17)Women:<15 min 1.0015–29 min 0.89 (0.78–1.02)30+ min 0.98 (0.88–1.09)Cardiovascular mortality:Men:<15 min 1.0015–29 min 1.08 (0.95–1.23)30+ min 1.05 (0.93–1.19)Women:<15 min 1.0015–29 min 0.78 (0.62–0.97)30+ min 0.97 (0.82–1.15)
**Barengo et al. (2005)**Eastern and South West Finland	Prospective Cohort Study to assess association between active travel and risk of hypertension; Participation: Men 73–79%; Women 83–85%. After exclusions for use of hypertensives (2433) and incomplete data (828) leaving 5935 men and 6227 women. Population survey using independent random sample conducted in 1982, 1985, 1992. Self-administered questionnaire: 1 week of activity and demographics, Measured by a nurse: blood pressure, height, weight.	Aged 25–6451% womenMean age: Men 43.5 (SD 8.6); Women 43.4 (SD8.5); BMI: Men 26.3 (SD3.5); Women 25.4 (SD4.4).Active travel: Men, Women<15 min:70, 53%15–29 min:16%,21%30+ min:14%, 26%	Adjusted hazard ratio (95% CI) for hypertensionMen:<15 min/day 1.0015–29 min/day 1.05 (0.86–1.29)30+ min/day 0.84 (0.67–1.05)Women:<15 min/day 1.0015–29 min/day 0.90 (0.69–1.17)30+ min/day 1.06 (0.85–1.34)Men and Women<15 min/day 1.0015–29 min/day 0.98 (0.84–1.16)30+ min/day 0.96 (0.82–1.12)
**Batty et al. (2001)**London, UK	Prospective Cohort Study to assess association between active travel and cause specific mortality. 12552 male participants. 16 men were missing travel information and 873 had non-comparable work grades so were excluded leaving 1163 for analysis.Workplace cohort survey in 1967 and 1969 measured: height, weight; blood pressure; lung function; cholesterol; glucose tolerance; questionnaire on demographics, health status and physical activity. Follow up = 25 years.	Aged 40–640% womenTravel activity:0–9 min: 19.6%;10–19 mins: 44.9%20+ mins: 35.5%	12 mortality endpoints but after adjusting for confounders there were no statistically significant differences between those who actively travelled more or less than 20 minutes on the (one-way) journey to work.
**Bere et al. (2011)**Rotterdam, Netherlands & Kristiansand, Norway	Prospective Cohort study to assess the relationship between cycling to school and weight status.890 participants at baseline, 890 completed two year follow up (54% participation).2 year follow up.Measurements at baseline and at follow up: questionnaire of demographics and travel mode, objective [dh]height and weight measures converted into BMI scores.	Secondary school studentsMean age 13.3 years at baseline.42% cycled on 3 or more days per week at baseline.	Odds Ratio (95% CI) of being overweight compared with the other groups:No cycling 1.05 (0.57,1.59)Started Cycling 1.22 (0.40,3.70)Stopped Cycling 3.19 (1.41,7.24)Continued to cycle 0.44 (0.21,0.88)
**Besson et al. (2008)**Norfolk, UK**Moayyeri et al. (2010)**	Prospective Cohort Study14905 participants at baseline,2 LTFU, 99.99% participationMedian follow up 7 years, total 102,964 person-yearsMeasurements at baseline:Self-completed questionnaire of how people travelled to work and for other journeys – responses converted into MET.h.wk^−1^Measured BMI, blood pressure, smoking status, alcohol consumption, social class, medical history of CVD & cancerAssessed association of different domains of physical activity with bone strength and fracture risk. 60.5% participation rate, 96% completion rate. Mean follow-up time 7.5 years. Measurements: self-completed questionnaire of previous years' physical activity behaviour and quantitative ultrasound assessment of the heel. Participants followed up through NHS database to health endpoints.	Men and women aged 45–79	For active travellers (>8 MET.h.wk^−1^):All cause mortalityHR 0.82 (0.67–1.00)Cardiovascular mortalityHR 0.79 (0.55–1.13)Adjusted Hazard ratios for any type of fracture and hip fracture were non-significant in both men and women, numbers of participants were small.
**Chillon et al. (2012)**Sweden**Cooper et al. (2008)**Odense, Denmark**Andersen et al. (2011)**Odense, Demark	Prospective Cohort Study to assess the effects of active travel on fitness, fatness and cardio-metabolic risk factors.907 participants at baseline, 60% drop out rate,262 participants (142 girls, 120 boys) had complete records at 6 year follow up.Measurements:Height, weight, waist circumference, skinfold thickness and pubertal status.Questionnaire about usual travel to school mode.Cycle ergometer cardio-respiratory fitness test measured maximal oxygen uptake.Blood pressure and blood samples for cholesterol, triglycerides and insulin.Prospective Cohort Study; Survey of a representative sample of children to measure the effects of cycling to school on cardio-respiratory fitness (CRF). 771 invited to participate from 25 schools in 1997, 589 (310 girls, 279 boys) consented. Follow up after 6 years in 2003 re-examined 384 (214 girls, 170 boys). Completion 64%. Measurements: height, weight, skinfold thickness and pubertal status. Questionnaire about usual travel to school mode and journey time. Cycle ergometer cardio-respiratory fitness test measured maximal oxygen uptake. Accelerometer measured physical activity.Prospective Cohort Study to assess effects of cycling to school on cardiovascular risk factors. For participants see above. 50 participants excluded, 334 (57%) completed the study.Measurements: Same as Chillon et al. above.	School ChildrenBaseline characteristicsBoys (SD) Girls (SD)Age 9.5 (0.4) 9.5 (0.4)BMI 17.2 (2.5) 17.1 (2.3)Walk 60% 49%Cycle 12% 13%Passive travel 28% 38%90% of cyclists reported journey time <15 minutes.Baseline characteristicsBoys (SD) Girls (SD)Age 9.7 (0.4) 9.6 (0.4)BMI 17.1 (2.0) 17.2 (2.5)Walk/Cycle 65.5% 65.3%88% of walkers and 95.5% of cyclists reported journey time <15 minutes.Same as Cooper above.	Children who cycled to school increased their fitness 13% more than those who used passive modes and 20% more than those who walked during the 6 year period.Children who took up cycling during the follow up period increased their fitness by 14% compared with those who did not.No significant association between travel mode to school and fatness or cardiometabolic risk factors.Cardio-respiratory fitness (CRF) was significantly higher among girls (0.33W kg-1P<.001) and boys (0.34WW kg-1 P = 001) who cycled to school at either the beginning or the end of the study compared with those who did not cycle at either time. CRF of those who stopped cycling was no different to those who never cycled. Cycling at both time points and taking up cycling were significant predictors of CRF in 2003.Passive travellers and walkers had similar cardiovascular risk measures and were combined for analysis as ‘non-cyclists’.At baseline there were differences in fitness levels between cyclists and non cyclistsAt follow up there were differences between cyclists and non-cyclists in TG, TC/HDL, fasting glucose, HOMA and sum of z-scores (P<0.05).Children who took up cycling during the follow up period were significantly fitter, had significantly lower waist circumference, glucose, insulin, HOMA, TC/HDL values and clustered risk scores compared with those who did not.
**Hayashi et al. (1999)**Osaka, Japan	Prospective Cohort Study; Workplace cohort survey to measure the association between duration of walk to work and risk of hypertension. Between 1981–1990 7979 enrolled; 1875 excluded because of hypertensionLeaving 6104 to participate but 87 were lost to follow up (1.4%) so full results only available for 6017 men; 99% completion.Measurements: Questionnaire of physical activity and lifestyle; Blood pressure; Fasting blood glucose; Follow up period 7–16 years.	Employees of a gas company with sedentary occupation.0% womenAged 35–60Age 41.7+/−6.5BMI 22.6+/−2.6	Number Needed to Walk: NNT 111.1 for 11–20 minute walk to work compared with less than 10 minute walk to work.NNT 26.3 (CI 26.1–26.5) for 21+ minutes walk to work compared with less than 10 minute walk to work.Adjusted relative risk of hypertension:0–10 min: 1.0011–20 min: 0.88 (0.75–1.04)21+ min: 0.70 (0.59–0.95)
**Heelan et al. (2005)**Nebraska, USA	Prospective Cohort Study; 600 children invited to participate;60% participation rate; 6.2% non-completers; Measurements at baseline and 6 months: Weight, height and Skinfold; self-administered questionnaire on travel mode to school.	ChildrenAged 10.2 (+/−0.7) years.56% girlsBMI at baseline 19.4 (+/−3.7)	After adjusting for baseline BMI the partial r = 0.03 P<0.05. For overweight children partial r = 0.10; P<0.05. For normal weight children, no significant relationship for BMI. No significant association between travel mode and body fat.
**Hu et al. (2003)**Eastern and South West Finland	Prospective Cohort Study; Random population sample survey to assess association between active travel and type 2 diabetes risk. Measurements: Self-administered questionnaire re: medical history, socioeconomic factors, smoking, physical activity, occupational, leisure time and commuting. Baseline surveys with cohorts in 1982,1987 and 1992, 74–88% participation rate; Mean follow up period = 12 years	Aged 35–6453% women	Adjusted relative risk for type 2 diabetes0 min: 1.00;1–29 min: 0.96 (0.74–1.25)>/ = 30 min 0.64 (0.45–0.92)
**Hu et al. (2005, 2007, 2007)**Finland	Prospective Cohort Study; To examine the association between active commuting and risk of coronary heart disease. Self-administered questionnaire surveys of smoking, socioeconomic, alcohol consumption, medical history, occupational, leisure time and commuting physical activity at baseline in cohorts in 1972, 1977, 1982, 1987, 1992 and 1997. 74–88% participation rate. Mean follow up = 18.9 years.	Aged 25–6452% women	Adjusted Hazard ratios of coronary heart disease:Men: 0 min: 1.00; 1–29 min: 0.99 (0.91–1.08); >/ = 30 min 0.99 (0.90–1.10); Women: 0 min 1.00; 1–29 min: 0.95 (0.83–1.08); >/ = 30 min 0.80 (0.69–0.92)
**Lofgren et al. (2010)**Malmo, Sweden	Prospective Cohort Study to assess whether active travel to school is associated with larger gain in bone mineral content and bone width than passive travel. 133 boys and 99 girls; 5 boys and 6 girls did not answer question on mode of transport so were excluded. 47 boys and 28 girls had no consistent mode of travel. So 39% boys and 34% girls were excluded before study began. 6% girls and 11% boys dropped out during study. 2 year follow up. Measurements taken at baseline and 2 years: Accelerometers worn for 4 days; Questionnaire on activity; bone mineral content.	Age 7–9 years75% girls	After adjustment there were no differences in annual changes in bone mineral content or bone width between children travelling actively or passively to school.
**Luoto et al. (2000)**Finland	Prospective Cohort Study; To assess the effect of active travel on breast cancer risk. Random sample of 30,548 women sent postal lifestyle questionnaire between 1978–1984, 1986–1993. Data then linked to cancer registry data. Response rate 75–86%	Aged 15–64100% women50%+ active commuters	No significant difference in breast cancer risk by travel mode. Adjusted Relative Risk (95% CI): Staying at home: 1.00; Passive travel: 0.94 (0.66–1.34); <30 mins/day 0.89 (0.67–1.18); >/ = 30 mins/day 0.87 (0.62–1.24)
**Matthews et al. (2007)**Shanghai, China	Prospective cohort study to assess association between active travel and all cause mortality. 93% participation rate; >99% completion rate. Mean follow up 5.7 years. Measurements:Interview re: activity in previous 5 years – exercise participation, household activities, active transport, occupational activity. Also, demographics, medical history, lifestyle behaviours, occupational history.	Aged 40–70100% women	Walking MET hours/day adjusted hazard ratio for all cause mortality.0–3.4 1.003.5–7.0 0.94 (0.81–1.09)7.1–10.0 0.83 (0.69–1.00)>/ = 10.1 0.86 (0.71–1.05)Cycling MET hours/day adjusted hazard ratio for all cause mortality:0 1.000.1–3.4 0.79 (0.61–1.01)>/ = 3.5 0.66 (0.40–1.07)
**Pabayo et al. (2010)**Quebec, Canada	Prospective Cohort Study; 1170 participants; 78% completed study (1170/1492);Measurements at baseline, 1 and 2 years:structured interview,height and weight measurement converted into BMI z-scores	ChildrenAged approximately 6 years.51.8% girls81.8% normal weight at baseline.	Children who used active travel from kindergarten (aged 6) to grade 2 (aged 8) had an average BMI z-score 0.3 (p = 0.003) standard deviations lower than other children. No significant associations between sustained active travel and relative weight.
**Rosenberg et al. (2006)**Southern California, USA	Prospective Cohort Study;1083 participants at baseline;85% participation, 924 completed all measurements.Measurements at baseline, 6, 12, 18 months:- Self-completed questionnaire on travel mode to school.- weight, height and skinfold. Accelerometers worn for 1 evening and the following morning (74% participation). Parents completed demographics survey (75% completion rate).	4^th^ grade pupils at elementary schools.46.8% girls	Change in BMI and skinfolds over the study period was not significantly different for children classified as active or passive travellers.
**Sato et al. (2007)**Kansai, Japan	Prospective cohort study;11,073 participants at baseline (87.6% participation rate), 77.4% completion rate.4 year follow up.Measurement – fasting plasma glucose, BMI, questionnaire of physical activity and lifestyle factors.	Sedentary employees aged 40–55 yearsMean age 47.8+/−4.20% women	Adjusted odds ratio of incidence of Type 2 diabetes:0–10 min:1.0011–20 min: 0.86 (0.70–1.06)21+ min: 0.73 (0.58–0.92)
**Wagner et al. (2001, 2002, 2003)**France & Northern Ireland	Prospective cohort study to assess association between risk of CHD event and active travel. 91% participation rate. 9% LTFUMeasurements: Interview re: health, lifestyle, socioeconomic data and physical activity; BMI , waist circumference; Blood sample	Aged 50–59 yearsMean age 54.9+/−2.90% womenBMI 26.6+/−3.4	Adjusted relative risk for CHD events 1.19 (0.81–1.76).

**Table 3:** Experimental and observational studies of active travel and health outcomes – summary of effects.

Author (Year) and Setting	Results
**Andersen et al. (2000)**Copenhagen County, Denmark	Relative risk of all-cause mortality:0.72 (95% CI 0.57–0.91) in cycle-commuters compared to non-cyclists.
**Barengo et al. (2004)**Eastern & South-west Finland	Adjusted hazard ratio (95% CI)All cause mortality:Men: Women:<15 min 1.00 1.0015–29 min 1.01 (0.92–1.11) 0.89 (0.78–1.02)30+ min 1.07 (0.98–1.17) 0.98 (0.88–1.09)Cardiovascular mortality:Men: Women:<15 min 1.00 1.0015–29 min 1.08 (0.95–1.23) 0.78 (0.62–0.97)30+ min 1.05 (0.93–1.19) 0.97 (0.82–1.15)
**Barengo et al. (2005)**Eastern and South West Finland	Adjusted hazard ratio (95% CI) for hypertensionMen and Women<15 min/day 1.0015–29 min/day 0.98 (0.84–1.16)30+ min/day 0.96 (0.82–1.12)
**Batty et al. (2001)**London, UK	No statistically significant differences between those who actively travelled more or less than 20 minutes on the (one-way) journey to work for 12 mortality endpoints after adjusting for confounders.
**Bere et al. (2011)**Rotterdam, Netherlands & Kristiansand, Norway	Odds Ratio (95% CI) of being overweight compared with the other groups:No cycling 1.05 (0.57,1.59)Started Cycling 1.22 (0.40,3.70)Stopped Cycling 3.19 (1.41,7.24)Continued to cycle 0.44 (0.21,0.88)
**Besson et al. (2008)**Norfolk, UK**Moayyeri et al. (2010)**	For active travellers (>8 MET.h.wk^−1^):All cause mortality: HR 0.82 (0.67–1.00)Cardiovascular mortality: HR 0.79 (0.55–1.13)No significant differences between those who travelled actively and those who did not for any type of fracture and hip fracture in either men or women.
**Chillon et al. (2012)**Sweden**Cooper et al. (2008)**Odense, Denmark**Andersen et al. (2011)**Odense, Demark	Children who cycled to school increased their fitness 13% more than those who used passive modes and 20% more than those who walked during the 6 year period.Children who took up cycling during the follow up period increased their fitness by 14% compared with those who did not.No significant association between travel mode to school and fatness or cardiometabolic risk factors.Cardio-respiratory fitness (CRF) was significantly higher among girls (0.33W kg-1P<.001) and boys (0.34WW kg-1 P = 001) who cycled to school at either the beginning or the end of the study compared with those who did not cycle at either time.CRF of those who stopped cycling was no different to those who never cycled.Cycling at both time points and taking up cycling were significant predictors of CRF in 2003.At follow up there were differences between cyclists and non-cyclists in cardiovascular risk factors: TG, TC/HDL, fasting glucose, HOMA and sum of z-scores (P<0.05).Children who took up cycling during the follow up period were significantly fitter, had significantly lower waist circumference, glucose, insulin, HOMA, TC/HDL values and clustered risk scores compared with those who did not.
**De Geus et al. (2007)**Oost-Vlaanderen, Belgium	Cycle commuting showed significant improvements in fitness after 12 weeks as measured by absolute and relative maximal power and maximal exhaustion.
**De Geus et al. (2008, 2009)**Oost-Vlaanderen, Belgium	Minutes and calories burned per week through all physical activity were higher in the intervention group than the control group (but not statistically significant for minutes in the second 6-month period).
**Hayashi et al. (1999)**Osaka, Japan	Number Needed to Walk:111.1 for 11–20 minute walk to work compared with less than 10 minute walk to work.26.3 (CI 26.1–26.5) for 21+ minutes walk to work compared with less than 10 minute walk to work.Adjusted relative risk:0–10 min: 1.0011–20 min: 0.88 (0.75–1.04)21+ min: 0.70 (0.59–0.95)
**Heelan et al. (2005)**Nebraska, USA	After adjusting for baseline BMI the partial r = 0.03 P<0.05.For overweight children partial r = 0.10; P<0.05.For normal weight children, no significant relationship for BMI.No significant association between travel mode and body fat.
**Hendriksen et al. (2000)**Amsterdam, Netherlands	No significant weight change in control or intervention group after 1 year.Maximal external power increased in the intervention group 13% in the first 6 months while it stayed the same in the control group.Maximal oxygen uptake – significant change in men only in intervention group in first 6 months.
**Hu et al. (2003)**Eastern and South West Finland	Adjusted relative risk for type 2 diabetes0 min: 1.00;1–29 min: 0.96 (0.74–1.25)>/ = 30 min: 0.64 (0.45–0.92)
**Hu et al. (2005, 2007, 2007)**Finland	Adjusted Hazard ratios of coronary heart disease:Men: Women:0 min: 1.00 1.001–29 min: 0.99 (0.91–1.08) 0.95 (0.83–1.08)>/ = 30 min 0.99 (0.90–1.10) 0.80 (0.69–0.92)
**Lofgren et al. (2010)**Malmo, Sweden	No differences, after adjustment, in annual changes in bone mineral content or bone width between children travelling actively or passively to school.
**Luoto et al. (2000)**Finland	No significant difference in breast cancer risk by travel mode.Adjusted Relative Risk (95% CI):Staying at home: 1.00;Passive travel: 0.94 (0.66–1.34);<30 mins/day: 0.89 (0.67–1.18);>/ = 30 mins/day: 0.87 (0.62–1.24)
**Matthews et al. (2007)**Shanghai, China	Walking MET hours/day adjusted hazard ratio for all cause mortality.0–3.4 1.003.5–7.0 0.94 (0.81–1.09)7.1–10.0 0.83 (0.69–1.00)>/ = 10.1 0.86 (0.71–1.05)Cycling MET hours/day adjusted hazard ratio for all cause mortality:0 1.000.1–3.4 0.79 (0.61–1.01)>/ = 3.5 0.66 (0.40–1.07)
**Mutrie et al. (2002)**Glasgow, UK	3 of 8 SF36 subscales significantly improved in the mean intervention group score compared with the control group:Mental Health (72 to 76 vs. 73 to 71);Vitality (57–64 compared with 61);General Health (71 to 76 vs 75 to 73)
**Oja et al. (1991, 1998)**Finland	Intervention vs control group:4.5% (p = 0.02) net increase in maximal oxygen uptake10.3% net increase in maximum treadmill time (p = <0.001)5% (p = 0.06) increase in HDL cholesterol.No significant changes in serum total cholesterol or triglyceride concentrations.No changes in bodyweight or leisure-time physical activity in either group.
**Pabayo et al. (2010)**Quebec, Canada	Children who used active travel from kindergarten (aged 6) to grade 2 (aged 8) had an average BMI z-score 0.3 (p = 0.003) standard deviations lower than other children.No significant associations between sustained active travel and relative weight.
**Rosenberg et al. (2006)**Southern California, USA	No significant difference in the change in BMI and skinfolds over the study period for children classified as active or passive travellers.
**Sato et al. (2007)**Kansai, Japan	Adjusted odds ratio of incidence of Type 2 diabetes:0–10 min: 1.0011–20 min: 0.86 (0.70–1.06)21+ min: 0.73 (0.58–0.92)
**Wagner et al. (2001, 2002, 2003)**France & Northern Ireland	Adjusted relative risk for CHD events 1.19 (0.81–1.76).

**Table 4:** Quality Assessment.

GROUP	PAPER	SELECTION BIAS	STUDY DESIGN	CONFOUNDERS	BLINDING	DATA COLLECTION	WITHDRAWALS/DROPOUTS	APPROPRIATE STATS	PERCENTAGE EXPOSED	CONSISTENCY	CONTAMINATION
Obesity Prospective Cohorts	**Bere** 2011	Strong	Weak	Moderate	Not Applicable	Strong	Weak	Yes	Not applicable	Yes	Yes
	**Heelan** 2005	Moderate	Weak	Weak	Not Applicable	Strong	Strong	Yes	Not applicable	Yes	Can't tell
	**Pabayo** 2010	Moderate	Weak	Weak	Not Applicable	Moderate	Moderate	Yes	Not applicable	Yes	Can't tell
	**Rosenberg** 2006	Strong	Weak	Weak	Not Applicable	Moderate	Strong	Yes	Not applicable	Yes	Can't tell
Other Health Outcomes Intervention Studies	**De Geus** 2007	Weak	Weak	Weak	Not Applicable	Strong	Strong	No	80–100%	Yes	Can't tell
	**De Geus** 2008 & 2009	Weak	Moderate	Moderate	Not Applicable	Strong	Strong	Yes	Less than 60%	Yes	Yes
	**Hendriksen** 2000	Weak	Strong	Weak	Not Applicable	Moderate	Strong	Yes	Not Reported	Yes	Can't tell
	**Mutrie** 2002	Weak	Strong	Weak	Not Applicable	Moderate	Weak	Yes	60–79%	Not Applicable	Can't tell
	**Oja** 1991 & 1998	Weak	Moderate	Weak	Not Applicable	Moderate	Strong	Yes	80–100%	Yes	No
Other Health Outcomes Prospective Cohorts	**Andersen** 2000	Moderate	Weak	Weak	Not Applicable	Moderate	Strong	Yes	Not applicable	No	Yes
	**Andersen** 2011	Moderate	Weak	Weak	Not Applicable	Strong	Weak	Yes	Not applicable	Yes	Yes
	**Barengo** 2004	Moderate	Weak	Weak	Not Applicable	Strong	Moderate	Yes	Not applicable	Yes	Yes
	**Barengo** 2005	Moderate	Weak	Weak	Not Applicable	Strong	Moderate	Yes	Not applicable	Yes	Yes
	**Batty** 2001	Weak	Weak	Strong	Not Applicable	Strong	Strong	Yes	Not applicable	Yes	Yes
	**Besson** 2008	Weak	Weak	Weak	Not Applicable	Moderate	Strong	Yes	Not applicable	Yes	Yes
	**Cooper** 2008	Moderate	Weak	Weak	Not Applicable	Strong	Moderate	Yes	Not applicable	No	Yes
	**Chillon** 2012	Moderate	Weak	Weak	Not Applicable	Strong	Weak	Yes	Not applicable	No	Yes
	**Hayashi** 1999	Weak	Weak	Strong	Not Applicable	Moderate	Strong	Yes	Not applicable	Yes	Yes
	**Hu** 2003	Moderate	Weak	Strong	Not Applicable	Strong	Strong	Yes	Not applicable	Yes	Yes
	**Hu** 2005, 2007 & 2007	Moderate	Weak	Weak	Not Applicable	Strong	Strong	Yes	Not applicable	Yes	Yes
	**Lofgren** 2010	Strong	Weak	Strong	Not Applicable	Moderate	Strong	Yes	Not applicable	No	Yes
	**Luoto** 2000	Moderate	Weak	Weak	Not Applicable	Weak	Strong	Yes	Not applicable	Yes	Yes
	**Matthews** 2007	Strong	Weak	Strong	Not Applicable	Moderate	Strong	Yes	Not applicable	Yes	Yes
	**Moayyeri** 2010	Moderate	Weak	Weak	Not Applicable	Strong	Strong	Yes	Not applicable	Yes	Yes
	**Sato** 2007	Strong	Weak	Weak	Not Applicable	Strong	Moderate	Yes	Not applicable	Yes	Yes
	**Wagner** 2001, 2002 & 2003	Strong	Weak	Strong	Not Applicable	Strong	Strong	Yes	Not applicable	Yes	Yes

### 1. Studies in adults

Eighteen studies in adults were identified; five intervention studies and thirteen prospective cohort studies.

#### 1.1 Intervention studies

The intervention studies included adults in north-west Europe and measured multiple health outcomes including fitness, blood pressure, cholesterol, oxygen uptake, and body weight [30], [31], [32], [33], [34], [35], [36]; none measured obesity directly. Three studies found improvements in fitness measures in the intervention group compared with the control group [30], [33], [35], [36], one found increased physical activity levels [31], [32], [37] but one did not [35], [36], two found no significant change in body weight [31], [32], [35], [36] and one found significantly higher scores for 3 of the 8 domains of the SF-36 in the intervention group [34]. All these studies were at risk of selection bias and none reported baseline differences between intervention and control groups for potential confounders [30], [31], [32], [33], [34], [35], [36], [37]. However, all five studies were rated moderately overall. All but one [30] were controlled with appropriate statistical analyses. All but one [34] had low levels of drop-out and ensured that the intervention was consistently applied.

#### 1.2. Prospective Cohort Studies

The 13 prospective cohort studies of adults (described below) [12], [13], [38], [39], [40], [41], [42], [43], [44], [45], [46], [47], [48], [49], [50], [51] covered a range of health outcomes. Eight were conducted in Scandinavia [12], [38], [39], [40], [42], [43], [44], [45], [46], [47]. This may reflect the longer history of higher population levels of active travel, as a result of which questions on active travel have been included in population surveys over recent decades. Overall, these studies reported conflicting findings when measuring similar mortality and cardiovascular outcomes, with the exception of diabetes where the 2 studies both found statistically significant positive results for active travellers compared with non-active travellers and hint at a dose-response relationship [43] [52].

Five studies investigated all cause mortality. One study in Denmark [38] found a significantly lower all-cause risk of mortality in cycle-commuters compared with non-cyclists - this was not found in a second such study in Finland [12]. Batty et al. (2001) [13] also found no statistically significant differences for 12 mortality endpoints between men in London, UK who actively travelled more or less than 20 minutes on their journey to work. Matthews et al. (2007) [48] studied women in China and found no significant relationship between walking and cycling for transport and all cause mortality [48]. Besson et al (2008) [53] studied men and women in Norfolk, UK and found a non-significant reduced risk of all cause mortality in those who travelled actively (measured as more than 8 metabolic equivalent task values (MET.h.wk^−1^)). None of these studies were rated consistently strong or moderate across all quality criteria. However they did all measure different levels of active travel among participants, which was a strength.

Five studies reported on cardiovascular outcomes. Besson et al.(2008) [53] found no significant reduction in cardiovascular mortality risk among active travellers whereas Barengo (2004) [12] in Finland found it to be significantly lower (adjusted hazard ratio 0.78 [CI: 0.62–0.97]) only among women actively travelling 15–29 minutes each way to work compared with those travelling less than 15 minutes each way but not in those travelling more than 30 minutes each way, and not in men. Hu et al (2005, 2007, 2007) [42], [44], [45], also measured Coronary Heart Disease and found a significant relationship in women who travelled 30+ minutes per day (0.80 [CI:0.69–0.92]) compared with those who did not travel actively at all. Like Barengo (2004) [12], they found no relationship between active travel and Coronary Heart Disease (CHD) in men. Barengo (2005) [39] found no difference in hypertension risk between those travelling more or less than 15 minutes each way to work. Hayashi et al. (1999) [41] found a statistically significant reduced risk of hypertension in those men in Osaka, Japan who walked 21 minutes or more to work compared with men who walked less than 10 minutes (adjusted relative risk 0.70 [CI: 0.59–0.95]). However, it was not clear from either of these papers how frequently the active travellers walked to work. Wagner et al. (2001, 2002, 2003) [49], [50], [51] found a statistically non-significant increase in risk of CHD events in men walking and cycling to work, although the amount of exercise taken while actively commuting was not recorded.

Four studies examined health outcomes other than all cause mortality or cardiovascular disease. Two studies found significant benefits of active travel for reducing diabetes risk. A study in Japan by Sato et al found a 27% reduced odds of type 2 diabetes among men who walked more than 21 minutes to work compared with those who walked less than 10 minutes (CI:0.58–0.92) [52]. A study in Finland [43] found the relative risk for Type 2 diabetes to be 34% lower among active travellers travelling 30 minutes or more per day compared with those not travelling actively (CI: 0.45–0.92). Luoto et al. 2000 [47] reported a non-significant reduction in relative breast cancer risk at 15 years follow-up of 0.87 (CI: 0.62–1.24) in women who actively travelled more than 30 minutes each day. Moayyeri et al. (2010) found no significant association between active travel and bone strength and fracture risk, but the numbers of study participants who travelled actively were extremely small [54].

### 2. Studies in children

No intervention studies in children were identified. Four prospective cohort studies were identified with obesity outcomes and two with other health outcomes.

#### 2.1 Obesity

One prospective cohort study measured the BMI of children aged 13 and again two years later in the Netherlands and Norway [55]. This study found that those children who continued to cycle to school throughout the study period were less likely (OR 0.44, 95% confidence interval 0.21,0.88) to be overweight than those who did not cycle to school, those who took up cycling and those who stopped cycling to school. Also those who stopped cycling to school during the study were more likely to be overweight than the other groups combined (OR 3.19, 95% confidence interval 1.41, 7.24). However the authors acknowledged that there were some limitations to this study including uncontrolled confounding variables and a relatively high dropout of 56% of participants between baseline and follow-up measurements. A study in Denmark and Sweden with six year follow-up of children from aged nine found no significant association between the obesity measures (BMI, skin-folds and waist circumference) and travel mode [56] [29]. Three other prospective cohort studies with obesity outcomes were all conducted in North America and included children aged ten years or younger at baseline who were followed up for between six months and two years [57], [58], [59]. BMI measurements were taken in all three studies and skinfold measurements were taken in two of the studies. There was no significant association between active travel and the obesity outcome measures in any of the studies. All three studies were rated low on the quality assessment measure as no data on baseline differences between groups were presented.

#### 2.2 Other health outcomes

Two studies examined health outcomes other than obesity. One study conducted in Denmark and Sweden found that children who cycled to school in Denmark had significantly better cardio-respiratory fitness [40] and cardiovascular risk markers than those who did not [56]. This study took a range of measures of school children aged 9 and repeated the measurements after six years. In Sweden, children who cycled to school increased their fitness 13% more than those who used passive modes and 20% more than those who walked during the six year period. Children who took up cycling during the follow up period increased their fitness by 14% compared with those who did no t [29]. However, no significant association between travel mode to school and cardiovascular risk factors was found in the Swedish arm of the study. Interestingly, the Danish arm of the study found that walkers had the same fitness levels as those who travelled by ‘passive’ modes [56]. While the study scored moderately well for selection bias (76% participation in Denmark), drop out from this study was 60% in Sweden and 43% in Denmark. This study, as was the case for many of the prospective cohort studies, may have been at risk of contamination or co-intervention as monitoring during the follow-up period was not reported. Lofgren et al. (2010) [46] also studied children actively travelling to school in Malmö, Sweden and measured a range of bone health indicators but found no significant relationship. This study scored relatively well in the quality assessment, with good controlling of confounders and high participation levels, although as with all the prospective cohort studies scored weak on study design.

## Discussion

This is the first review to bring together all prospective observational and intervention studies to give an overview of the evidence on health effects of active travel in general. Previous systematic reviews of health outcomes of active travel have included primarily cross-sectional studies from which reliable inferences about causality cannot easily be drawn, or have relied on indirect evidence on the effects of physical activity on health, as opposed to the effects of active travel. Although we found no prospective studies of active travel with obesity as a primary outcome in adults, and no significant associations between obesity and active travel in studies which included children, for other health outcomes small positive health effects were found in groups who actively travelled longer distances including reductions in risk of all cause mortality [38], hypertension [41], and in particular Type 2 diabetes [43], [52].

One challenge to synthesising and using this evidence is that “active travel” is not defined consistently across studies, and the definition is dependent on what is considered normal in a particular setting. For example Luoto (2000) [47], and Barengo (2004, 2005) [12], [39] considered active travel to be more than 30 minutes per day and inactive travel to be less than 30 minutes per day. Batty (2001) [13], Sato (2007) [52] and Hayashi (1999) [41] however considered active travel to be more than 20 minutes per day. Differences in health outcomes between people who actively travel 29 minutes per day and those who travel 31 minutes per day are unlikely, so differences between active and sedentary populations may be masked by the methods by which active travel is defined and reported. Meanwhile Besson (2008) [53] and Moayyeri (2010) [54] considered active travel to be more than 8 metabolic equivalent task (MET) hours per week while Matthews (2007) [48] considered it to be more than 3.5 metabolic equivalent task hours per day which may reflect differences in norms between UK and China in terms of active travel.

In light of this, users of the findings of this and similar reviews need to consider the extent to which we can generalise between studies conducted in different countries or settings. In particular, the amount of exertion required to travel actively may be greater in some settings than others for the same journey time, due to differences in congestion, terrain and climate. In countries where current levels of physical activity are low (such as the UK, where only 39% of men and 29% of women achieve 30 minutes of moderate intensity physical activity of any type five times a week [60] [61]) adding 30 minutes of active travel per day might well produce much larger changes in health at a population level than were measured in non-UK studies. The prospective cohort studies also tended to focus on travel to work or school rather than active travel for general transportation, which again may limit generalisability.

The study by Cooper et al. (2008) [40] of school children in Odense, Denmark found that 65% of boys and girls walked or cycled to school, a much higher proportion than is currently found in the UK. However, journey times were less than 15 minutes for the majority of active travellers so the health effects of active travel for such short periods are difficult to measure in isolation. This highlights one of the difficulties of assuming active travel to school in young people to be a major source of physical activity, as it is common for children only to walk or cycle to school when the journey time is relatively short. In adults as little as 10 minutes of physical activity are acceptable to contribute to their weekly physical activity target of minimum 150 minutes. However children aged five – 18 are expected to be physically active for a minimum of 420 minutes per week [8] so a short active commute to school will not make a significant contribution to their overall physical activity requirements. The study by Lofgren et al. [46] included a study population with fairly high levels of physical activity overall and half the participants were active travellers, which makes it difficult to attribute health outcomes to active travel alone, as active travel may not contribute significantly to participants overall physical activity levels.

De Geus et al. (2007) [30] highlighted one of the difficulties of measuring active travel in intervention studies as they found that study participants cycled 13% faster when their fitness was being measured compared to their usual speed on their daily cycle commute. The process of measuring active travel can therefore result in an over-estimate of the health benefits conferred by active travel. It is also not clear whether levels of active travel impact on levels of other types of physical activity such as sport and leisure. This relationship has been explored by, among others, Dombois et al who found no relationship between levels of sports activity and mode of travel in adults in the Swiss Alps [62], and also by Santos et al who found a more complex relationship between different types of activity in children in Portugal [63]. Thus issues including type of terrain, problems of definition, study design and the difficulty of disentangling the effects of active travel from more general physical activity make synthesis difficult.

There is a particular challenge in measuring health outcomes in children because some health outcomes relating to physical activity can take many years to develop. For example an intervention study by Sirard et al. involving children in the USA measured moderate-to-vigorous physical activity (MVPA) in a randomised controlled trial with 12 participants and a two week duration [37]. However, it could not be included in this review because it did not measure a health outcome.

This review also highlights the difficulty in measuring health outcomes of active travel in the general population. In prospective cohort studies if the follow-up period is short then it may not be possible to measure health effects that take many years to appear. Conversely in those studies which do have long follow-up periods of many years there is the risk that active travel has not been consistently adhered to throughout the follow up period.

The likelihood of health outcomes will depend on the context within which individuals are travelling – length of journey, frequency of travel, nature of the terrain, risk of injury, levels of air pollution and so on as well as other aspects of the lifestyles of the participants. For example travelling actively may mean that the individual is more or less likely to be physically active at other times, or they may modify their diet. It may mean that they are more or less likely to strengthen social networks. It is also important to note that active travel not only potentially benefits health by way of physical activity but may also off-set air pollution from motorised vehicles and contribute to social and environmental goals such as improving social cohesion and reducing CO_2_ emissions. These combined benefits are a potent argument for promoting active travel, and emphasise the importance of models which incorporate both health and non-health benefits [64], [65] such as carbon dioxide emissions.

Finally, designing searches which are both sensitive and specific is a challenge for public health systematic reviews. It is interesting to note that over 70% of the studies we identified were initially found through hand-searching, although some subsequently appeared in the database searches, which highlights the importance of a broad search not confined to electronic sources. While it is possible that studies may have been missed, our comprehensive search for studies makes it unlikely that a significant body of work has been excluded.

## Conclusions

While the studies identified in this review do not enable us to draw strong conclusions about the health effects of active travel, this systematic review of intervention and prospective studies found consistent support for the positive effects on health of active travel over longer periods and perhaps distances, and it is of interest that there is some evidence that active travel may reduce risk of diabetes. This may be an important area for future research.

These cautious conclusions on the health impact of active travel do not, of course, mean that now is the time to confine active travel to the walk from the front door to the car door. The evidence on the effect of physical activity is sufficiently strong to suggest that the part played by active travel is well worth maintaining. Other aspects of active travel, including a reduction in pollution, and in carbon footprint are clear potential co-benefits and likely to become even more so.

## Supporting Information

Appendix S1 **PRISMA flowchart.**
(DOCX)

Appendix S2 **PRISMA checklist.**
(DOCX)

Appendix S3 **Search strategy.**
(DOCX)
