# Supplementary material for: What Are the Health Benefits of Active Travel? A Systematic Review of Trials and Cohort Studies
Source: PLoS One. 2013 Aug 15;8(8):e69912. doi: 10.1371/journal.pone.0069912 (PMC3744525; doi:10.1371/journal.pone.0069912)
Supplement: Appendix S1 — PRISMA flowchart. (DOCX) [file pone.0069912.s001.docx]

**S 1 PRISMA Flow Diagram for Active Travel Revi**

35 full text articles excluded

4 articles identified

32 papers reporting 24 studies included in qualitative synthesis

21842 records identified through database searching

213 records identified through other sources

63 full-text articles assessed for eligibility

19055 records excluded

19118 records screened

19118 records after duplicates removed
