# Supplementary material for: What Are the Health Benefits of Active Travel? A Systematic Review of Trials and Cohort Studies
Source: PLoS One. 2013 Aug 15;8(8):e69912. doi: 10.1371/journal.pone.0069912 (PMC3744525; doi:10.1371/journal.pone.0069912)
Supplement: Appendix S3 — Search strategy. (DOCX) [file pone.0069912.s003.docx]

**S 3 Search Strategy**

The below search strategy was used in Medline. No time, topic or language exclusions or limits were applied. This was the template search strategy which was adapted as needed to fit the other 10 databases searched for this review. The exact search for each of the databases is available on request from the authors.

*Search in ‘keyword’*

*1. (Walk* or cycle* or cycli* or bicycle* or bike* or walking bus* or ecological commut* or ecological transport* or non-auto* or non-motori?e*).mp.
2. (green* adj3 (travel* or transport* or commut*)).mp.
3. ((activ* or health*) adj3 (travel* or transport* or commut*)).mp.
4. (ecological adj3 (travel* or transport* or commut*)).mp.
5. or/1-4*

*6.(Prospective adj1 (study or studies)).mp*

*7.(Program evaluation or evaluation research).mp.*

*8.(randomi$ or randomly).mp.*

*9.(controlled adj2 (trial or trials or study or studies or experiment$)).mp*

*10.(before adj1 after).ab,ti.*

*11.(control adj1 group$).ab,ti.*

*12.(intervention adj1 group$).ab,ti.*

*13.(experimental adj1 group$).ab,ti.*

*14.(comparison adj1 group$)ab,ti.*

*15. Or/6-14*

*16.(Body mass index or BMI or body mass gain or overweight or weight gain or weight status or weight management or weight loss or body weight or waist circumference or obesity or calorie* or energy)*

1. *5 and 15 and 16*
2. *17 in humans*
